# Comparisons of different neoadjuvant chemotherapy regimens with or without stereotactic body radiation therapy for borderline resectable pancreatic cancer: study protocol of a prospective, randomized phase II trial (BRPCNCC-1)

**DOI:** 10.1186/s13014-019-1254-8

**Published:** 2019-03-27

**Authors:** Suizhi Gao, Xiaofei Zhu, Xiaohan Shi, Kai Cao, Yun Bian, Hui Jiang, Kaixuan Wang, Shiwei Guo, Huojun Zhang, Gang Jin

**Affiliations:** 1 Department of Surgery, Changhai Hospital Affiliated to Navy Medical University, 168 Changhai Road, Shanghai, 200433 China; 2 Department of Radiation Oncology, Changhai Hospital Affiliated to Navy Medical University, 168 Changhai Road, Shanghai, 200433 China; 3 Department of Radiology, Changhai Hospital Affiliated to Navy Medical University, Shanghai, 200433 China; 4 Department of Pathology, Changhai Hospital Affiliated to Navy Medical University, Shanghai, 200433 China; 5 Department of Gastroenterology, Changhai Hospital Affiliated to Navy Medical University, Shanghai, 200433 China

**Keywords:** Neoadjuvant, Stereotactic body radiation therapy, Chemotherapy, S-1, Gemcitabine, Borderline resectable pancreatic cancer

## Abstract

**Background:**

Few patients with pancreatic cancer may be candidates for immediate surgical resection at the initial diagnosis. Even if patients with borderline resectable pancreatic cancer (BRPC), micrometastases may occur before surgery. Therefore, neoadjuvant therapy is vital for improved survival, which has been confirmed in previous studies that neoadjuvant chemotherapy with or without radiotherapy provides superior overall compared with upfront surgery. However, question of whether the addition of radiotherapy to neoadjuvant chemotherapy can improve prognosis compared with chemotherapy alone is a challenging matter. Moreover, most of previous studies only adopted conventional radiotherapy as the neoadjuvant modality though stereotactic body radiation therapy (SBRT) has been proven effective and commonly employed in pancreatic cancer. Also, no studies have evaluated the efficacy of S-1 as the neoadjuvant chemotherapy regimen for BRPC albeit similar prognosis has been found between S-1 and gemcitabine in advanced pancreatic cancer. Hence, the aim of this study is to investigate whether neoadjuvant chemotherapy plus SBRT results in better outcomes compared with neoadjuvant chemotherapy alone and also compare the efficacy of gemcitabine plus nab-paclitaxel with SBRT and S-1 plus nab-paclitaxel with SBRT.

**Methods:**

Patients with biopsy and radiographically confirmed BRPC, no prior treatment and severe morbidities are enrolled. They will be randomly allocated into three groups: neoadjuvant gemcitabine plus nab-paclitaxel, neoadjuvant gemcitabine plus nab-paclitaxel with SBRT and neoadjuvant S-1 plus nab-paclitaxel with SBRT. Standard doses of gemcitabine and nab-paclitaxel are used. The radiation dose of SBRT is 7.5-8Gy/f for 5 fractions. Surgical resection will be performed 3 weeks after SBRT. Artery first approach pancreaticoduodenectomy or radical antegrade modular pancreatosplenectomy will be used for the tumor in the head or body and tail of the pancreas, respectively. The primary endpoint is overall survival. The secondary outcomes are disease free survival, pathological complete response rate, R0 resection rate and incidence of adverse effects.

**Discussion:**

If results show the survival benefits of neoadjuvant chemotherapy plus SBRT and similar outcomes between S-1 and gemcitabine, it may provide evidence of clinical practice of this modality for BRPC.

**Trial registration:**

The study has been registered in ClinicalTrial.gov (NCT03777462).

## Introduction

Pancreatic cancer has been the fourth cause of cancer mortality in the US with a 5-year survival rate of 3% [1]. In the recent release of cancer statistics of GLOBALCAN database, pancreatic cancer is the seventh leading cause of cancer death in both males and females worldwide [2], and it has been projected that pancreatic cancer will surpass breast cancer as the third cause of cancer death in the European Union [3]. Slightly increasing incidences and cancer mortality of pancreatic cancer were also found in China [4].

Though surgical resection is considered as the only curative intent treatment, the 5-year survival rate of patients with upfront surgery is less than 20% [5]. Therefore, it may be implied that patients could not benefit from surgery alone even with early stage pancreatic cancer at the initial diagnosis, which might be attributable to the micrometastases invisible in the imaging examinations before surgery. As a result, optimal multimodality is in demand for reducing the risk of micrometastases before operations and the incidences of positive or narrow margins, especially for borderline resectable pancreatic cancer (BRPC). Although it has still remained controversial whether neoadjuvant treatment should be performed for BRPC in the guidelines, a recent study has shown survival benefits with neoadjuvant chemoradiation compared with upfront surgery [6].

Due to high intolerance and incidences of adverse effects of FOLFIRINOX in Chinese, other regimens with mild toxicities may be the alternatives. It has been proven that gemcitabine plus nab-paclitaxel contributed to increased survival in pancreatic cancer [7]. And this modality has been included as an option for neoadjuvant chemotherapy in the guideline though there is limited evidence to recommend specific regimens. Moreover, nab-paclitaxel has been confirmed to bind secreted protein acidic and rich in cysteine in the tumor, which resulted in accumulation of chemotherapy drugs and degradation of mesenchymal tissues, thus probably improving the efficacy of chemotherapy. In addition to gemcitabine and nab-paclitaxel, S-1, the prodrug of 5-fluorouracil comprising of tegafur, gimeracil and oteracil, was also an option as the regimen. Previous studies have proven that S-1 was not inferior to gemcitabine in terms of overall survival (OS) rates and disease free survival (DFS) rates with tolerable effects [8, 9]. Hence, nab-paclitaxel based chemotherapy may contribute to synergic effects with S-1.

Furthermore, compared with conventional radiotherapy, the advantages of stereotactic body radiation therapy (SBRT), precise treatment delivery by motion compensation strategies with high doses to target volumes, abrupt dose fall-off outside target volumes, shorter durations and real-time tracking, has rendered SBRT as an appealing technique of radiotherapy, which has been widely employed for pancreatic cancer with high rates of local control and acceptable toxicities. Nevertheless, only conventional radiotherapy was employed in the neoadjuvant treatment in the previous studies.

Therefore, the aim of the study is to compare and evaluate the efficacy of neoadjuvant gemcitabine plus nab-paclitaxel versus gemcitabine plus nab-paclitaxel with SBRT versus S-1 plus nab-paclitaxel with SBRT for BRPC.

## Methods

### Objectives

The primary objective is to compare the OS of patients receiving neoadjuvant gemcitabine plus nab-paclitaxel versus gemcitabine plus nab-paclitaxel with SBRT versus S-1 plus nab-paclitaxel with SBRT for BRPC. The secondary objective is to compare DFS, pathological complete response rates and adverse effects of the three groups.

### Hypothesis

Neoadjuvant chemotherapy plus SBRT is superior to neoadjuvant chemotherapy alone regarding OS, while no significant difference is found between gemcitabine plus nab-paclitaxel with SBRT and S-1 plus nab-paclitaxel with SBRT.

### Study design, setting and participants

This is a randomized, prospective, single center and phase III trial. Patients aged above 18, with radiographically and biopsy proven BRPC and without any prior treatment will be screened for eligibility. Therefore, all potential patients would receive fine needle aspirations guided by endoscopic ultrasound. Details about the inclusion and exclusion criteria are shown in Table 1. Additionally, though there are difficulties in defining this borderline resectable pancreatic cancer due to heterogeneity of definitions of this disease entity proposed by different institutions, it is likely that most of studies would employ the definition demonstrated in the NCCN guideline. Therefore, the definition of BRPC is referred to that clarified in the NCCN guideline (Table 2).

**Table 1:** Inclusion and exclusion criteria

Inclusion criteria	Exclusion criteria
• Age ≥ 18 years old and ≤ 80 years old	• Metastatic pancreatic cancer
• Histological proven pancreatic adenocarcinoma	• Patients who had surgeries, chemotherapy or other treatments before inclusion
• Borderline resectable pancreatic cancer proven by imaging examinations via multidisciplinary approaches according to NCCN guidelines	• Impaired organ functions: A. Heart failure (New York Heart Association III–IV), coronary heart disease, myocardial infarction within 6 months, severe cardiac arrhythmia B. Respiratory failure
• No prior chemotherapy or radiotherapy	• Confirmed other cancer within 5 years
• ECOG of 0 or 1	• Pregnant women or lactating women
• Routine blood test: absolute neutrophil count> 1500/mm^3^, platelet> 100,000/mm^3^	• Patients enrolled in other clinical trials or incompliant of regular follow up
• Normal liver function: serum total bilirubin≤2 .0mg/dl, ALT and AST < 2.5 times of the upper limit of normal value	• Patients who did not provide an informed consent
• Normal kidney function: serum creatinine< 1.5 times of the upper limit of normal value or creatinine clearance rate > 45 ml/min	
• No severe comorbidities

**Table 2:** The definition of borderline resectable pancreatic cancer in the NCCN guideline

Resectability status	Arteral	Venous
Borderline resectable	Pancreatic head/unicinate process: • Solid tumor contact with CHA without extension to CA or hepatic artery bifurcation allowing for safe and complete resection and reconstruction. • Solid tumor contact with the SMA of ≤180° • Solid tumor contact with variant arterial anatomy and the presence and degree of tumor contact should be noted if present, as it may affect surgical planning. Pancreatic body/tail: • Solid tumor contact with the CA of ≤180° • Solid tumor contact with the CA of > 180° without involvement of the aorta and with intact and uninvolved gastroduodenal artery thereby permitting a modified Appleby procedure	• Solid tumor contact with the SMV or PV of > 180°, contact of ≤180° with contour irregularity of the vein or thrombosis of the vein but with suitable vessel proximal and distal to the site of involvement allowing for safe and complete resection and vein reconstruction. • Solid tumor contact with the inferior vena cava.

**Fig. 1:**
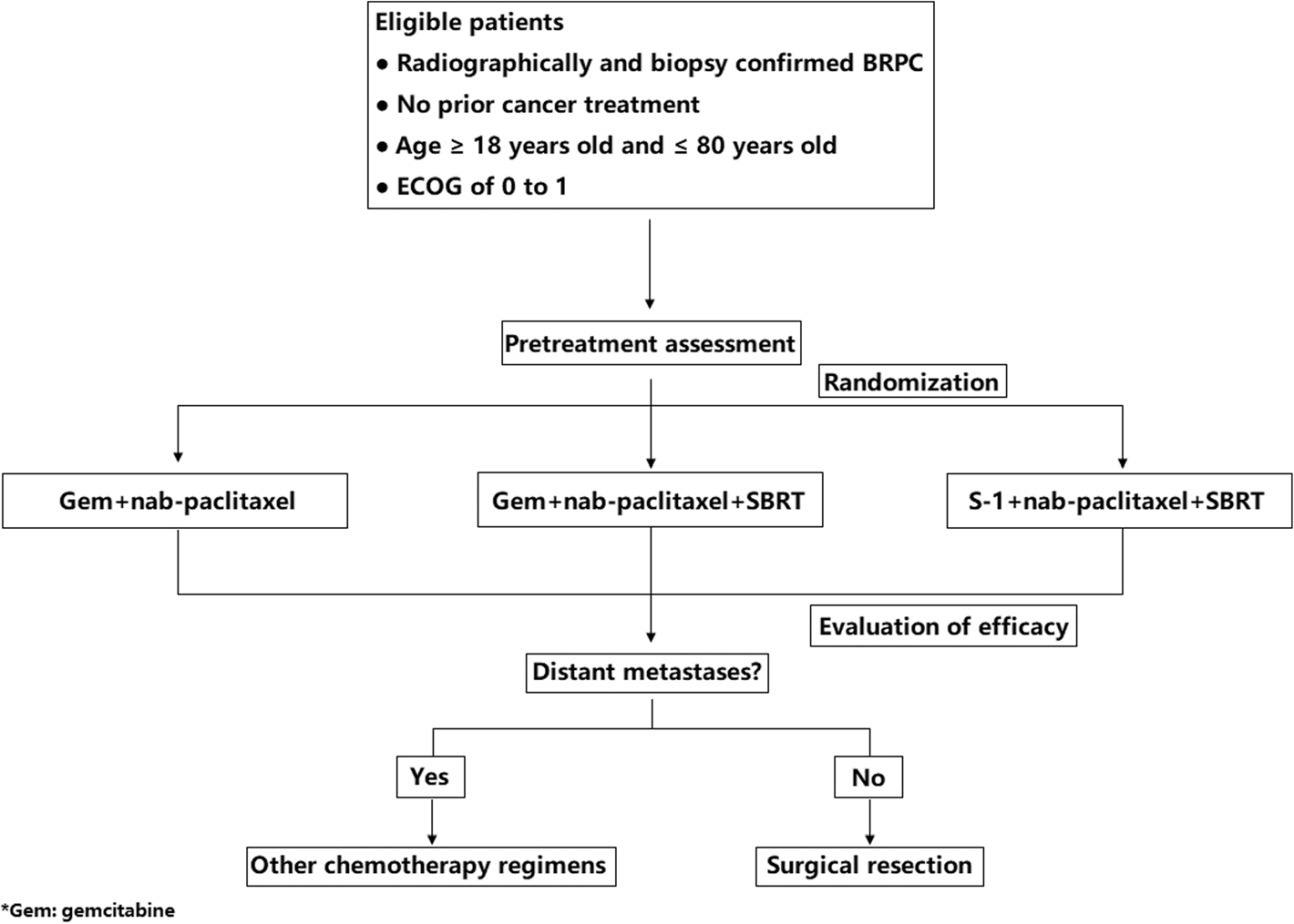
Flow diagram of the study

Eligible participants would receive personal interviews with physicians for a detailed explanation of the whole study and related treatment. It is mandatory to acquire the written informed consents before the study. Afterwards, patients are required to complete the pretreatment evaluations, including blood routine tests, liver and renal function tests, coagulation function tests, serum tumor marker (CA19–9) tests, enhanced CT and MRI, circulating cell free DNA (cfDNA), circulating tumor cells (CTC), DNA sequences of the specimens from fine-needle aspirations and pancreatic cancer-derived organoid cultures with specimens. In each group, participants will be randomized with a 1:1:1 allocation to receive gemcitabine plus nab-paclitaxel or gemcitabine plus nab-paclitaxel and SBRT or S-1 plus nab-paclitaxel and SBRT. Randomizations will be performed by a computer-generated random numbers table. After completion of the whole chemotherapy, patients will first receive PET-CT to exclude distant metastases and then undergo SBRT. Those with distant metastases will be precluded from the study and receive chemotherapy continuously. Likewise, patients confirmed with distant metastases after chemotherapy and SBRT will receive other aggressive chemotherapy regimens rather than surgical resections (Fig. 1).

### Ethics

The study is performed in accordance with the Declaration of Helsinki. All patients will be enrolled only after comprehensive information concerning the nature, scope, and possible consequences of the clinical trial has been provided to them in an understandable way by the physicians. Written informed consent will be obtained from each patient before the enrolment. During the study, details about the procedures, benefits and risks of chemotherapy, SBRT and surgery will be elucidated for those patients.

All physicians and patients involved in the study will be blinded to the allocations. Only researchers not involved in the study will be responsible for the randomization procedures. Additionally, patients could withdraw from the study at any time for any reason without any consequences. Clinicians are also required to record any adverse effects promptly in case that the treatment may be stopped temporarily or patients may be excluded from the study due to chemotherapy or radiotherapy induced toxicities at the physicians’ discretion. Those dropping out of the study would receive other alternative treatment based on multidisciplinary approaches.

The protocol has been reviewed and approved by the institutional review board of our hospital (CHEC2018–176). The study has also been registered in ClinicalTrial.gov (NCT03777462).

### Intervention

All participants are allocated into the three groups randomly. Intravenous administration of gemcitabine (1000 mg/m^2^) or nab-paclitaxel (125 mg/m^2^) is initiated on day 1, 8 and 15 during each 4-week cycle, which will repeat for 3 cycles. S-1 is orally administrated at a dose of 80 mg/m^2^ for 18 days followed by a 10-day rest, which also continues for 3 cycles.

SBRT is performed after completion of the chemotherapy. The protocol of SBRT is similar to our previous studies [10–13]. Patients are required to be on fasting for 4–6 h before CT simulation. Vacuum bags are used for immobilization during SBRT. SBRT is delivered via Cyberknife, an image-guided frameless stereotactic robotic radiosurgery system (Accuray Corporation, Sunnyvale, California, USA). Before the simulation, fiducials should be implanted adjacent to the tumor guided by endoscopic ultrasound. Therefore, motion management during treatment would be performed by Synchrony® Respiratory Tracking System. A plain CT and an enhanced pancreatic parenchymal CT are performed for radiation treatment planning and target delineations. The scan range includes the whole pancreas, at least 10 cm above and below the tumor, with a slice thickness of 1 .5mm. Gross tumor volume (GTV) is delineated as a radiographically evident gross disease by the enhanced CT acquired from the portal-venous phase. At the discretion of the physician, clinical target volume (CTV) encompassing areas of the potential subclinical disease spread is also designated. In most cases, the CTV equals to GTV. A 2–5 mm expansion margin is included to determine the planning target volume (PTV). When tumors are adjacent to the critical organs, the expansion of GTV should be avoided at this direction. At least 90 % of PTV should be covered by the prescription dose. The prescribed dose is 7.5-8Gy/f for 5 fractions. Dose constraints of normal tissues are referred to the American Association of Physicists in Medicine guidelines in TG-101 [14].

Surgical resection is performed 3 weeks after SBRT. For the surgical approach of the tumor in the head of the pancreas, artery first approach pancreaticoduodenectomy is employed [15–17]. Regarding the tumor in the body or tail of the pancreas, radical antegrade modular pancreatosplenectomy (RAMPS) is used [17, 18].

### Data collection

The schematic diagram for the timeline of data collections and evaluations of efficacy and safety is shown in Table 3. Pretreatment assessment will be performed and recorded by physicians. After randomizations, efficacy and toxicity of chemotherapy and SBRT and post-operative complications will also be evaluated and recorded by the clinicians. Furthermore, all information about the randomizations and data about the pretreatment assessment and follow-up will be firstly carefully checked by the physicians and re-checked by the researchers not involved in the study to promote data accuracy and completeness. And data entry into the database will be performed by the researchers.

**Table 3:** Schematic diagram for the schedule of enrolment, interventions, and assessments

Time point	Enrolment	Allocation	Post-allocation								
			During chemotherapy with or without SBRT				Post-surgery				
			1 month	2 months	3 months	4 months	1 month	3 months	6 months	1 year	2 years
Enrolment											
Eligibility screen	X										
Informed consent	X										
Allocation		X									
Interventions											
Gem+nab-paclitaxel											
Gem+nab-paclitaxel+SBRT											
S-1 + nab-paclitaxel+SBRT											
Assessments											
Physical examinations	X		X	X	X	X	X	X	X	X	X
Laboratory tests	X		X	X	X	X	X	X	X	X	X
Imaging examinations	X			X		X		X	X	X	X
cfDNA, CTC ^a^	X					X^b^					
Organoid	X						X				
DNA sequencing of specimens	X						X				
Adverse effects			X	X	X	X	X	X			
Surgical safety							X	X			
Survival status										X	X

^a^Conducted if recurrence occurs besides those performed in the above time points

^b^Conducted after completion of neoadjuvant therapy and before surgery

The investigators will not assume any demands, including publishing or reporting of individual patient’s data, especially data required for this clinical trial, until a valid consent has been obtained. Patients’ data would be kept strictly confidential within the study, but their pseudonymous medical records and information would be extracted from the database and reviewed for trial purposes by authorized individuals other than their treating physicians.

### Follow-up

Patients are required to receive regular follow-up 1 month, 3 months, 6 months, 1 year and 2 years after surgery. In each follow-up, patients will undergo physical examinations (ECOG, evaluations of symptoms and weight) and laboratory tests (including blood tests, liver and kidney functions, serum tumor biomarker). Imaging examinations will be performed 3 months, 6 months, 1 year and 2 years after surgery to evaluate efficacy. Post-operative complications would be evaluated 1 month and 3 months after surgical resection. Adverse effects of chemotherapy will be assessed according to The Common Terminology Criteria for Adverse Events version 4.03 (CTCAE v4.03). Radiation-induced acute toxicities are adverse effects that occur within 90 days after treatment, and determined by the Radiation Therapy Oncology Group, ‘Acute radiation morbidity scoring criteria’. If patients experience disease progression during follow-up, cfDNA and CTC would be performed to evaluate the tumor burden and identify genetic differences of the tumor cells before and after treatment as far as possible. Disease progression is determined by the Response Evaluation Criteria in Solid Tumors (RECIST) criteria (v1.1).

### Outcomes

The primary outcome is OS and 1-year and 2-year OS rate. OS is defined as the time interval from the time of surgical resection to the date of all-cause death or the last follow-up.

The secondary outcomes are DFS, pathological complete response rate, R0 resection rate, incidence of adverse effects. DFS is determined as the time interval from the date of surgery to the confirmation of recurrence at any sites or death from any cause, if this occurs before disease progression, or the last follow-up. The definition of pathological complete response is ypT0N0M0, which is no evidence of invasive tumor in the pancreas and the draining lymph nodes at the time of surgery. R0 resection is defined as both clear macroscopic and microscopic margins (≥1 mm).

### Sample size and statistical analysis

Assuming, for the gemcitabine plus nab-paclitaxel group, a median 2-year OS rate of about 20%, we target a 10 and 15% relative improvement in 2-year OS rate for the group allocated to S-1 plus nab-paclitaxel with SBRT and gemcitabine plus nab-paclitaxel with SBRT over the gemcitabine plus nab-paclitaxel group, respectively. This would provide approximately 80% power with one-sided α of 0.025. Hence, the maximum number of required patients in each group is 73. Thereafter, when a 10% dropout rate is taken into account, the number of participants in each group is 81.

For the continuous variables, statistical normality will be assessed firstly. Comparisons between these main effects of the three groups will be then performed by analysis of variance or its’2 non-parametric equivalents as appropriate. Categorical data will be compared by Chi-square tests or Fisher’s Exact tests. The OS and DFS curves will be conducted by Kaplan-Meier method. Log-rank test will be employed for comparisons of OS and DFS. Proportional hazard Cox regression adjusted for variables will be used when necessary. Data analysis will be performed on both intention-to-treat and per-protocol basis. All *P* values will be reported using a significance level of 0.05.

## Discussion

It has been long suggested that upfront surgery is given first priority for patients with BRPC or resectable pancreatic cancer based on NCCN guidelines. However, recent studies have clarified the possibility of employment of neoadjuvant chemotherapy with or without radiotherapy, which may provide potential survival benefits. For resectable pancreatic cancer, systemic reviews indicated that neoadjuvant therapy was associated with improved OS [19–21]. Likewise, a recent study also identified that total neoadjuvant FOLFIRINOX followed by chemoradiotherapy brought about high rates of R0 resection and prolonged OS and DFS for patients with BRPC [22]. Furthermore, a meta-analysis has shown that neoadjuvant chemoradiotherapy appeared to improve OS compared with upfront surgery [23]. Therefore, it might be implied that neoadjuvant chemoradiotherapy could be an alternative to the surgery-first paradigm for BRPC albeit without high-level evidence. And in 2018 ASCO meeting, a randomized trial reported that preoperative chemoradiotherapy resulted in high rates of R0 resection, longer disease free survival, distant metastases free survival and locoregional recurrence free survival and a trend of superior OS compared with immediate surgery.

Additionally, in most of previous studies, conventional radiotherapy was used as the modality. Usually, radiation doses of 45-54Gy in 1.8–2.0Gy fractions are prescribed leading to a long treatment period, which may result in micrometastases during neoadjuvant therapy. For patients with BRPC, relatively small tumor volumes without obvious invasions of adjacent vessels may provide opportunities for dose-escalation. Hence, SBRT may be an appropriate choice due to its delivery of high biological effective doses within a short period and quick dose fall-off, which may not delay subsequent treatment and provide high pathological complete response rates with acceptable toxicities simultaneously, combined with chemotherapy. Though it might be implied from the previous studies that neoadjuvant chemotherapy with or without radiotherapy could be an alternative to the surgery-first paradigm for BRPC, potential benefits from neoadjuvant chemoradiotherapy still need to be confirmed in prospective studies. Therefore, the BRPCNCC-1 trial, a randomized and prospective study, investigates whether neoadjuvant chemotherapy plus SBRT can prolong survival for BRPC compared with neoadjuvant chemotherapy alone.

Though S-1 has been confirmed effective in pancreatic cancer, especially for Asians, most of studies have only adopted S-1 in advanced pancreatic cancer. No studies have evaluated the efficacy of S-1 in neoadjuvant therapy. Besides, only FOLFIRINOX or gemcitabine plus nab-paclitaxel is recommended in guidelines. As a result, it is necessary to assess whether neoadjuvant S-1 plus nab-paclitaxel with SBRT could be an alternative to neoadjuvant gemcitabine plus nab-paclitaxel with SBRT regarding OS.

Another concern about the trial is the definition of resectability status. Recently, a new international consensus on the definition and criteria of BRPC has been proposed [24]. It has been advocated that tumor biomarker, CA19–9, and performance status should be taken into account in addition to anatomical definition. In this consensus, CA19–9 level of less than 500 U/ml was also a pivotal prerequisite for BRPC. Moreover, patients with resectable pancreatic cancer and ECOG of 2 or more were defined as BRPC. In our study, all included patients have ECOG of less than 2. Additionally, CA19–9 may be a potential factor predictive of OS albeit no inclusion criteria about determinations of the CA19–9 level in our study. Therefore, regarding the two factors, prognosis would be compared between different levels of these factors in the subgroup analyses based on the new biological criteria for BRPC.

In summary, this pilot study, BRPCNCC-1, will investigate whether the addition of SBRT to neoadjuvant chemotherapy results in superior survival compared with neoadjuvant chemotherapy alone and compare the efficacy of S-1 plus nab-paclitaxel combined with SBRT and gemcitabine plus nab-paclitaxel combined with SBRT. The results may provide sufficient evidence for clinical practice of neoadjuvant therapy for BRPC.

## Abbreviations

BRPC: Borderline resectable pancreatic cancer
cfDNA: Circulating cell free DNA
CTC: Circulating tumor cells
CTV: Clinical target volume
DFS: Disease free survival
GTV: Gross tumor volume
OS: Overall survival
PTV: Planning target volume
RAMPS: Radical antegrade modular pancreatosplenectomy
SBRT: Stereotactic body radiation therapy
